# Free Osteoarticular Metatarsal Autograft in the Reconstruction of Giant Cell Tumor of Metacarpal: Report of Two Cases and Description of a Technique *

**DOI:** 10.1055/s-0041-1729942

**Published:** 2021-08-13

**Authors:** Luiz Fabiano Gomes Gularte

**Affiliations:** 1 Programa de Pós-Graduação em Saúde e Comportamento, Universidade Católica de Pelotas Pelotas, Rio Grande do Sul, RS, Brasil

**Keywords:** bone transplantation, giant cell tumor of bone, metacarpal bones, metacarpus

## Abstract

Giant cell tumors are benign but locally aggressive bone neoplasms containing many multinucleated giant cells similar to osteoclasts. The author reports the case of two patients with giant cell tumor in the metacarpals, one of whom was multicentric. Giant cell tumor in the hand is a rare condition, and, at this location, it commonly presents at an advanced stage, with extensive bone destruction.

Thus, its safe resection, associated with a large resulting bone failure, represents a great challenge to the orthopedist. The various treatment options described in the literature cause severe cosmetic and/or functional impairment to the hand. Thinking about it, the author describes the treatment technique through the transfer of metatarsus-free osteoarticular graft to the metacarpal with good functional and cosmetic results.

## Introduction

Giant cell tumors (GCTs) are benign but locally aggressive bone neoplasms characterized by a richly vascularized tissue containing many multinucleated giant cells similar to osteoclasts, and two types of proliferative mononuclear stromal cells, round and fusiform. 1 Round mononuclear cells, together with osteoclast-like giant cells, are reactive specialized benign cells derived from monocytes, and are recruited into the tumor by fusiform mononuclear cells, which are believed to be the only true neoplastic cells in GCTs. 2 3 These tumors are relatively uncommon, representing about 5% of all primary bone tumors 3 4 and about 22% of benign bone tumors. 5 They occur predominantly after skeletal maturity, exhibit a slight predominance in females, and have their peak incidence between 20 and 45 years of age. 4 6 About half of the cases occur around the knee, 7 being rare in the bones of the hand, 4 especially in skeletally-immature individuals, with few cases described in the literature. 8 Only 10.9% of the cases occur in patients older than 50 years of age. 5 Tumors of the metacarpals (MCs), metatarsus and phalanges are usually purely lytic and extend to the end of the bones, 9 and, when they reach the bones of the hands, they commonly present at an advanced stage, with extensive bone destruction, thus complicating their treatment. 10 Therefore, the reconstruction of voluminous lesions in the hand represents a great challenge, with a great risk of sequelae, amputation of rays, or deformities.

The aim of the present article is to describe the MC reconstruction technique after GCT resection using a free osteoarticular metatarsus autograft through the report of two cases. Case 1 is that of a 14-year-old female patient with a rapidly-growing GCT in the second left MC. Case 2 is that of a 62-year-old male patient with slower-growing multicentric CGT, with gradual worsening of right-hand pain and function over the course of the last 5 years, located in the fifth MC and fifth ipsilateral metatarsus.

## Surgical Technique

**Fig. 1:**
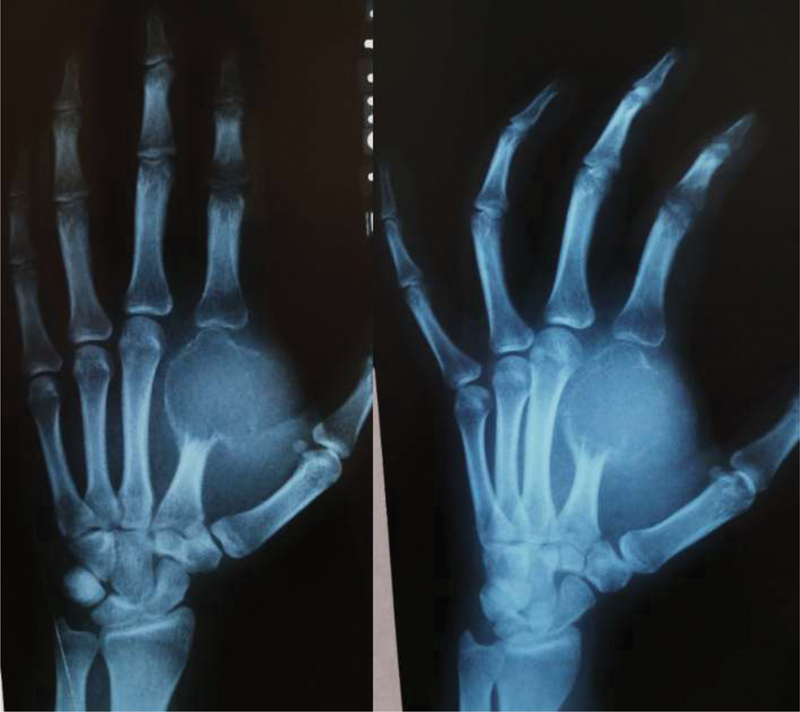
Case 1: frontal and oblique radiographs of the left hand two months after incisional biopsy by an oncologic surgeon. Notice that the exaggerated opening of the pseudocapsule enabled the rapid expansion of the tumor to the soft tissues.

To describe the surgical technique, we will use as an example the patient in Case 1 ( Fig. 1 ).

**Fig. 2:**
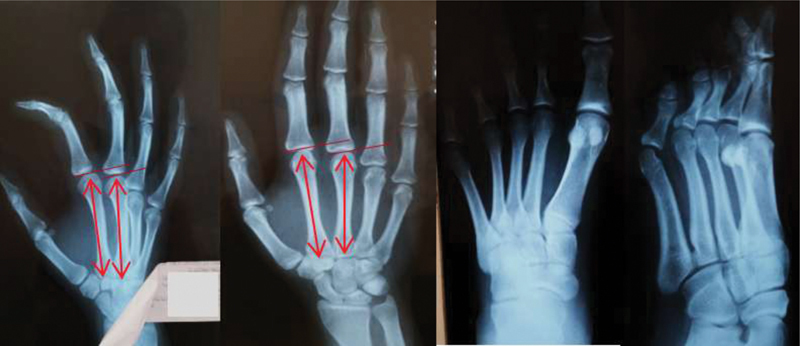
Case 1: radiographs of the right hand (normal) and left foot (ipsilateral to the lesion) to determine the length of the second metacarpal (MC) and its relationship with the third MC, in addition to verifying which metatarsus presented greater anatomical similarity to the second MC.

**Fig. 3:**
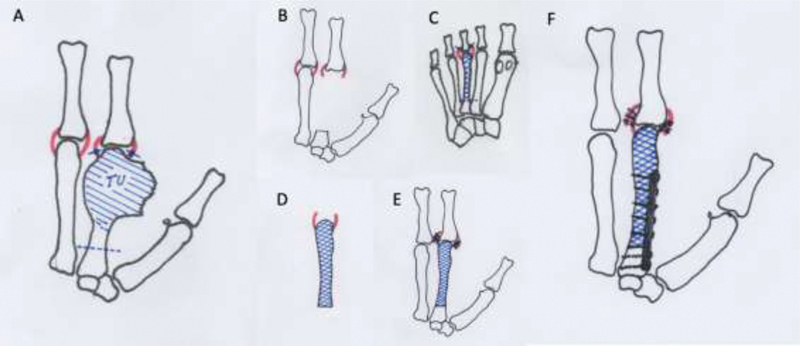
Scheme of the tumor resection and reconstruction technique with metatarsus-free osteoarticular graft. ( **A** ) The dashed line demarcates the location of the osteotomy, the full lines demarcate the joint capsule section (TU = tumor mass). ( **B** ) Hand after tumor resection, in which the base of the MC and the remaining capsular ligaments can be seen. ( **C** ) Choice of a metatarsus more similar in anatomy to the MC; the dashed line demarcates the location of the proximal osteotomy, and the full lines, the site of resection of the capsular ligaments. ( **D** ) Free metatarsus with its capsular insertions. ( **E** ) Suture of capsular ligaments. ( **F** ) Osteosynthesis with plate of minifragments.

**Fig. 4:**
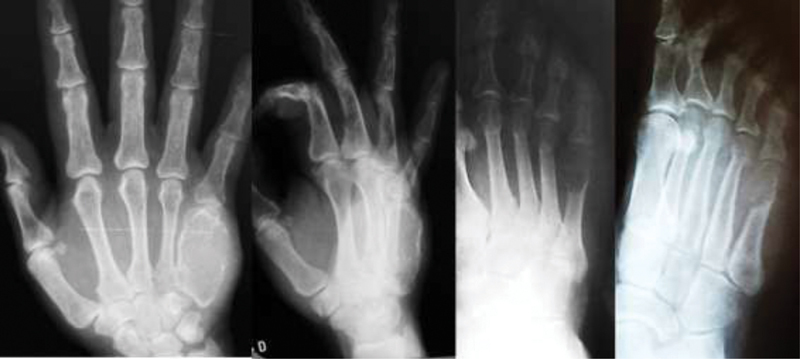
Case 2: right frontal and oblique radiographs of the right hand and foot, evidencing an expansive osteolytic lesion compromising the entire fifth right MC and the distal metaepiphyseal region of the fifth right metatarsus.

**Fig. 5:**
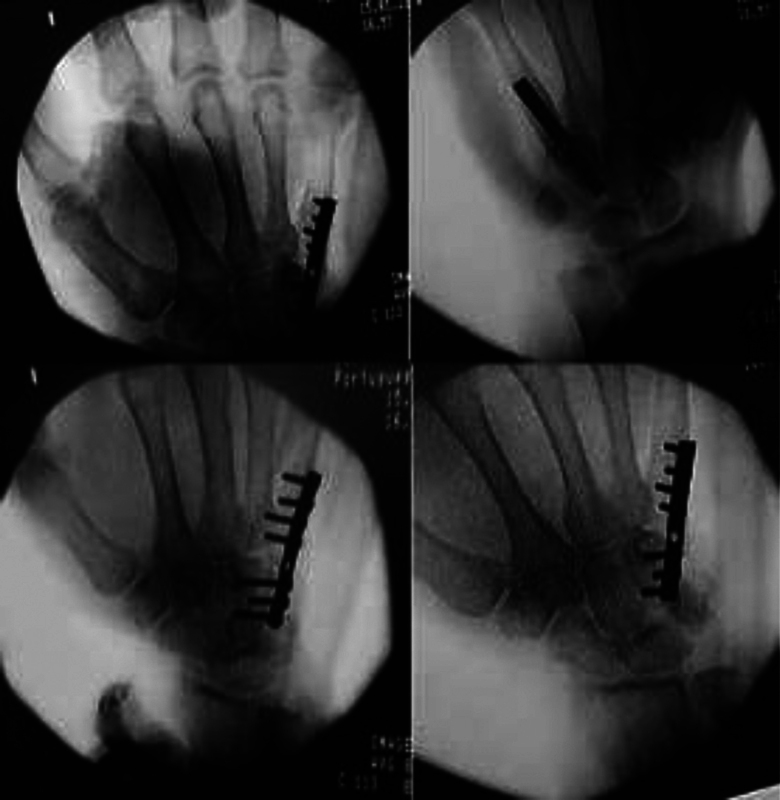
Case 2: anteroposterior, posteroanterior, and oblique radiographs in the immediate postoperative period of tumor resection and reconstruction with Free osteoarticular metatarsus autograft.

The objective criteria to choose the ideal metatarsus are the shape and diameter of the distal epiphysis and the width of the diasphysis more similar to the MC to be resected. Initially, a contralateral hand radiograph was performed to establish the relationships of the normal anatomy of the MCs, verifying the length of the second MC and the size relative to the third MC, in addition to radiographs of the ipsilateral foot to determine which metatarsus most resembled the second MC ( Fig. 2 ). Then, after osteotomy of the base of the second MC, the tumor was resected en bloc together with the biopsy scar, taking care to preserve as much as possible the metacarpophalangeal joint capsule. After tumor resection, the third ipsilateral metatarsus was collected through osteotomy at the base, taking care to completely remove the soft tissues around it, but preserving as much as possible the ligaments of the metatarsal-fallageal joint capsule. Osteosynthesis was performed with a plate of minifragments and suture of the remaining ligaments of the joint capsule of the second MC and the third metatarsus ( Fig. 3 ). After the procedure, the patient was immobilized with antebrachio-digital plaster, with the wrist at 20° to 30° of extension, the metacarpophalangeal joints at 70° to 90° of flexion, and the interphaplastic joints in total extension for 4 weeks. Then, physiotherapy of the hand was maintained, on average 3 times a week, and functional orthosis was performed by an occupational therapist, immobilizing the wrist and the metacarpophalangeal joint of the second finger in the 6 subsequent months, until signs of consolidation and radiological signs of increased graft porosity were observed, which are suggestive of graft revascularization. Fig. 4 and Fig. 5 show respectively the pre- and postoperative images of the patient in Case 2, in whom tumor resection and transfer of the third contralateral metatarsus were performed.

### Final comments

**Fig. 6:**
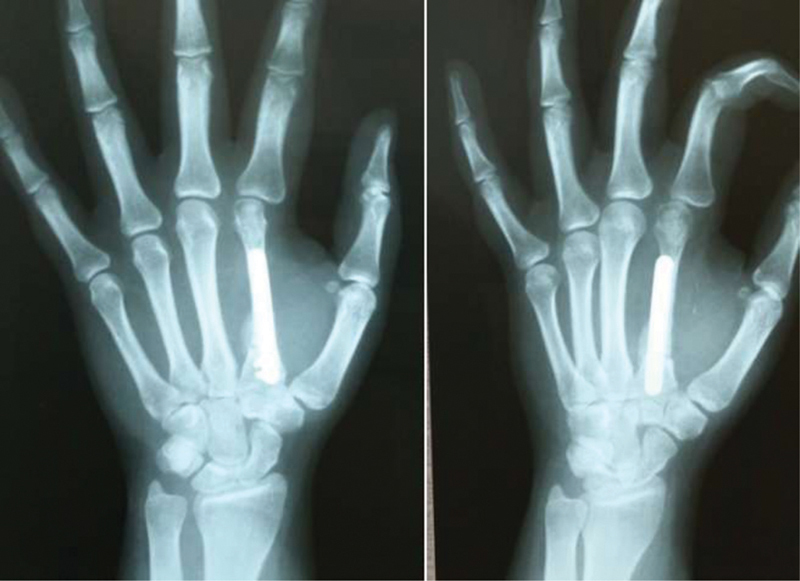
Patient in case 1 with 8.5 months after tumor resection and reconstruction with free osteoarticular metatarsus autograft. Radiograph 75 days after the iliac graft, showing complete consolidation and integration.

After six months of evolution, the osteotomy line showed no signs of consolidation between the graft and the host MC, which is why autologous iliac grafting was performed, evolving with complete bone consolidation in the eighth month ( Fig. 6 ). Currently, 5.5 years after the resection, the patient shows no signs of local recurrence, with function considered excellent according to the MSTS (Musculoskeletal Tumor Society). The male patient presented excellent evolution from the immediate postoperative period until the last revision two months postoperatively, when he returned to his hometown and was lost to follow-up.

None of the patients presented any complaint of foot pain or gait alteration after metatarsus removal. Thus, we conclude that the transfer of the metatarsal osteoarticular ligament complex for the reconstruction of MC defects after resection of giant cell tumors is a safe and efficient procedure, with good functional and cosmetic results in a medium-term follow-up.
