# Supplementary material for: Free Osteoarticular Metatarsal Autograft in the Reconstruction of Giant Cell Tumor of Metacarpal: Report of Two Cases and Description of a Technique
Source: Rev Bras Ortop (Sao Paulo). 2021 Aug 13;59(4):e622–5. doi: 10.1055/s-0041-1729942 (PMC11374390; doi:10.1055/s-0041-1729942)
Supplement: Related texts

# Research article: Enxerto osteoarticular livre de metatarso em reconstrução de tumor de células gigantes de metacarpo: Relato de dois casos e descrição de técnica

**Keywords:** metacarpo, ossos metacarpais, transplante ósseo, tumor de células gigantes do osso

## Abstract

Os tumores de células gigantes são neoplasias ósseas benignas, mas localmente agressivas, que contêm muitas células gigantes multinucleadas semelhantes aos osteoclastos. O autor relata o caso de dois pacientes com tumor de células gigantes nos metacarpos, dos quais um era multicêntrico. O tumor de células gigantes na mão é uma condição rara e, nesta localização, comumente apresenta-se em um estágio avançado, com extensa destruição óssea.

Dessa maneira, sua ressecção de forma segura, associada a uma grande falha óssea resultante, representa um grande desafio ao ortopedista. As várias opções de tratamento descritas na literatura ocasionam severa perda estética e/ou funcional da mão. Pensando nisso, o autor descreve a técnica de tratamento por meio da transferência de enxerto osteoarticular livre de metatarso para o metacarpo, com bons resultados funcionais e estéticos.

## Introdução

Os tumores de células gigantes (TCGs) são neoplasias ósseas benignas, mas localmente agressivas, caracterizadas por um tecido ricamente vascularizado, que contém muitas células gigantes multinucleadas semelhantes aos osteoclastos, e dois tipos de células estromais mononucleares proliferativas, as redondas e as fusiformes. 1 As células mononucleares redondas, juntamente com as células gigantes semelhantes aos osteoclastos, são células benignas especializadas, reativas, derivadas dos monócitos, e são recrutadas para o tumor pelas células mononucleares fusiformes, as quais acredita-se que sejam as únicas células neoplásicas verdadeiras no TCG. 2 3 Esses tumores são relativamente incomuns, e representam cerca de 5% de todos os tumores ósseos primários, 3 4 e cerca de 22% dos tumores ósseos benignos. 5 Ocorrem predominantemente após a maturidade esquelética, exibem uma leve predominância no sexo feminino, e têm o seu pico de incidência entre 20 e 45 anos de idade. 4 6 Cerca de metade dos casos ocorre em volta do joelho, 7 sendo raros nos ossos da mão, 4 sobretudo em indivíduos esqueleticamente imaturos, com poucos casos descritos na literatura. 8 Somente 10,9% dos casos ocorrem em pacientes acima dos 50 anos de idade. 5 Tumores dos metacarpos (MCs), metatarsos e falanges geralmente são puramente líticos, estendem-se até a extremidade dos ossos, 9 e, ao atingirem os ossos das mãos, comumente apresentam-se em um estágio avançado, com extensa destruição óssea, o que, portanto, complica o seu tratamento. 10 Por esse motivo, a reconstrução de lesões volumosas na mão representa um grande desafio, com grande risco de sequelas, amputação de raios, ou deformidades.

O objetivo deste artigo é descrever a técnica de reconstrução de MCs após a ressecção de TCG utilizando enxerto livre de matatarso, por meio do relato de dois casos. O caso 1 é o de uma paciente do sexo feminino de 14 anos com TCG no segundo MC esquerdo, de crescimento rápido. O caso 2 é o de um paciente do sexo masculino de 62 anos com TCG multicêntrico de crescimento mais lento, com piora gradual da dor e função da mão direita ao longo dos últimos 5 anos, localizado no quinto MC e no quinto metatarso ipsilateral.

## Técnica Cirúrgica

Para a descrição da técnica cirúrgica, utilizamos como exemplo a paciente do caso 1 ( Fig. 1 ).

**Fig. 1:** Caso 1: radiografias frontal e oblíqua da mão esquerda dois meses após a realização de biópsia incisional por cirurgião oncológico. Perceba que a abertura exagerada da pseudocápsula permitiu a rápida expansão do tumor para as partes moles.

Os critérios objetivos para a escolha do metatarso ideal são a forma e o diâmetro da epífise distal e a largura da diáfise mais semelhantes ao MC a ser ressecado. Inicialmente, foi realizada radiografia da mão contralateral para estabelecer as relações da anatomia normal dos MCs, verificando o comprimento do segundo MC e o tamanho relativo ao terceiro MC, além de radiografias do pé ipsilateral para determinar qual metatarso se assemelhava mais ao segundo MC ( Fig. 2 ). A seguir, após osteotomia da base do segundo MC, o tumor foi ressecado em bloco juntamente com a cicatriz da biópsia, tomando-se o cuidado de preservar ao máximo a cápsula articular metacarpo-falângica. Após a ressecção do tumor, foi realizada a coleta do terceiro metatarso ipsilateral com osteotomia na base, tomando-se o cuidado de retirar completamente as partes moles ao seu redor, mas mantendo o máximo dos ligamentos da cápsula articular metatarso-falângica. Foi realizada osteossíntese com placa de minifragmentos e sutura dos ligamentos remanescentes da cápsula articular do segndo MC e do terceiro metatarso ( Fig. 3 ). Após o procedimento, a paciente foi imobilizada com tala gessada antebraquio-digital com punho entre 20° e 30° de extensão, articulações metacarpo-falângicas entre 70° e 90° de flexão, e articulações interfalângicas em extensão total por 4 semanas. Depois, foi mantida com fisioterapia da mão, em média 3 vezes na semana, e órtese funcional confeccionada por terapeuta ocupacional imobilizando punho e a articulação metacarpo-falângica do segundo dedo nos 6 meses subsequentes, até que fossem observados sinais de consolidação, e que se percebessem sinais radiológicos de aumento da porosidade do enxerto, sugestivos da revascularização. A Fig. 4 e a Fig. 5 mostram, respectivamente, as imagens pré e pós-operatórias do paciente do caso 2, no qual foi realizada a ressecção do tumor e transferência do terceiro metatarso contralateral.

**Fig. 2:** Caso 1: radiografias da mão direita (normal) e do pé esquerdo (ipsilateral à lesão) para determinação do comprimento do segundo metacarpo (MC) e de sua relação com o terceiro MC, além de verificação de qual metatarso apresentava maior semelhança anatômica com o segundo MC.

**Fig. 3:** Esquema da técnica de ressecção do tumor e reconstrução com enxerto osteoarticular livre de metatarso. ( **A** ) A linha tracejada demarca o local da osteotomia, e as linhas contínuas demarcam local da secção da cápsula articular (TU = massa tumoral). ( **B** ) Mão após a ressecção tumoral, na qual se vê a base do MC e os ligamentos capsulares remanescentes. ( **C** ) Escolha do metatarso anatomicamente mais semelhante ao MC; a linha tracejada demarca o local da osteotomia proximal, e as linhas contínuas, o local da ressecção dos ligamentos capsulares. ( **D** ) Metatarso livre com suas inserções capsulares. ( **E** ) Sutura dos ligamentos capsulares. ( **F** ) Osteossíntese com placa de minifragmentos.

**Fig. 4:** Caso 2: radiografia frontal e oblíqua de mão e pé direitos evidenciando lesão osteolítica expansiva comprometendo todo o quinto MC direito e a região metaepifisária distal do quinto metatarso direito.

**Fig. 5:** Caso 2: radiografias anteroposteriores, posteroanteriores e oblíquas no pós-operatório imediato de ressecção do tumor e reconstrução com enxerto osteoarticular livre de metatarso.

## Comentários finais

Após seis meses de evolução, a linha de osteotomia não mostrava sinais de consolidação entre o enxerto e o MC hospedeiro, razão pela qual foi realizada enxertia autóloga de ilíaco, que evoluiu com completa consolidação óssea no oitavo mês ( Fig. 6 ). Atualmente, 5,5 anos após a ressecção, ela se encontra sem sinais de recidiva local, com função considerada excelente de acordo com o MSTS (Musculoskeletal Tumor Society). O paciente masculino apresentou excelente evolução do pós-operatório imediato até a última revisão com dois meses de pós-operatório, quando retornou à sua cidade natal e perdemos seu seguimento.

**Fig. 6:** Paciente do Caso 1 8,5 meses após a ressecção do tumor e a reconstrução com enxerto osteoarticular livre de metatarso. Radiografia com 75 dias de pós-operatório do enxerto de ilíaco, mostrando completa consolidação e integração.

Nenhum dos pacientes apresentou qualquer queixa de dor nos pés ou alteração da marcha após a retirada dos metatarsos. Dessa maneira, concluímos que a transferência do complexo ligamentar osteoarticular metatarsal, para a reconstrução de defeitos metacarpais após ressecção de tumores de células gigantes, é um procedimento seguro e eficiente, com bons resultados funcionais e estéticos num seguimento de médio prazo.
